# The Seroepidemiology of *Strongyloides stercoralis* Infection in Jamaica

**DOI:** 10.1155/2024/4578159

**Published:** 2024-11-09

**Authors:** Anique Vanessa Chin, Tamara Thompson, Cobrini S. Denton, John F. Lindo

**Affiliations:** ^1^Department of Microbiology, The University of the West Indies, Kingston 7, Jamaica; ^2^Department of Medicine, The University of the West Indies, Kingston 7, Jamaica; ^3^Department of Basic Medical Sciences, The University of the West Indies, Kingston 7, Jamaica; ^4^University Hospital of the West Indies, Kingston 7, Jamaica

**Keywords:** Jamaica, rapid ELISA, seroepidemiology, seropositivity, seroprevalence, *Strongyloides stercoralis*, strongyloidiasis

## Abstract

*Strongyloides stercoralis* is a soil-transmitted helminth which is distributed predominately throughout tropical and subtropical regions and is considered a neglected tropical disease. Due to low larval output, traditional microscopic methods lack sensitivity, especially in areas of low endemicity. Serological assays present an opportunity to study the epidemiology of *S. stercoralis* in areas of low endemicity such as Jamaica. The current study evaluated the seroprevalence of *S. stercoralis* in a selected subpopulation in Jamaica. An analysis was conducted on 311 archived serum samples previously submitted for investigating viral infections during a fever epidemic between 2014 and 2015. Randomly selected, anonymized sera were tested for the presence of *S*. *stercoralis* IgG antibodies using the AccuDiag *Strongyloides* IgG ELISA Kit. Data including age, sex, clinical diagnosis, and the geographic location of sample submission were recorded to delineate trends in demographic variables. The seropositivity rate of *S. stercoralis* was 15.43%. The rate among females and males was 16.45% and 14.47%, respectively (*χ*^2^ = 0.2339, *p*=0.629). The highest rate was found in middle adulthood (31–50 years) (26.53%; 13/49). The seroprevalence of *S. stercoralis* was significantly highest in a rural Regional Health Authority (33.33%; 14/42) and least within an urban Health Authority (9.71%; 17/175). Exposure to *S. stercoralis* appears to be highest in the rural Regional Health Authorities with an island-wide exposure rate of 15.43%. The rapid ELISA testing method for the detection of IgG antibodies to *S. stercoralis* used in this study may be useful as part of a combined approach to elucidate the epidemiology of this soil-transmitted parasite in Jamaica.

## 1. Introduction

*Strongyloides stercoralis*, a soil-transmitted helminth is the etiological agent of strongyloidiasis in the Caribbean [1–3]. The distribution of this parasite is global; however, endemic regions predominate in tropical and subtropical territories, where other common soil-transmitted helminth species including *Trichuris trichiura*, *Ascaris lumbricoides*, and hookworms are present [4, 5]. Together, these are important neglected tropical diseases and are major causes of morbidity, whereas there are diagnostic tools for rapid assessment of *T. trichiura*, *A. lumbricoides*, and hookworm infections, and *S. stercoralis* present diagnostic challenges due to the small number of larvae shed in stool.

The estimated global prevalence of strongyloidiasis is approximately 30–100 million people infected, according to the World Health Organization (WHO) [6]. This is arguably an underestimation, with some authorities quoting a prevalence of at least 370 million infected persons worldwide [7]. The root of this underestimation is innately related to the parasite's low larval output, which renders routine microscopy methods insensitive, limiting epidemiological studies. Integrating highly sensitive diagnostic methods to detect light and latent cases may be a step toward elucidating the epidemiology of *S. stercoralis* infections to determine the need for control measures.

The prevalence of *S. stercoralis* infection in highly endemic settings shows variability based on the mode of laboratory testing performed and the population being surveyed. In a systematic literature review of studies performed in Latin America over 3 decades, the prevalence of *S. stercoralis* was reported by Buonfrate et al. [7] to be > 20% in five countries—Argentina, Ecuador, Peru, Brazil, and Venezuela [7]. However, the majority of these surveys relied on direct fecal examination methods for laboratory detection of the parasite [7]. More sensitive diagnostic tools for the detection of *S. stercoralis* infection, such as serology, were less commonly used; hence, the resultant prevalence estimates were deemed to be low and not reliable [7].

The prevalence of *S. stercoralis* infection in the Caribbean has been sparsely reported. Ketzis et al. [1] reviewed prevalence data in 17 Caribbean countries, determining a range of < 1%–20.3% for 14 locations. Importantly, noted from this analysis was a reduction in prevalence over the past 40 years in the Caribbean, but not at the same rate of decline as other soil-transmitted helminths [1]. A similar trend in the decline in the prevalence of infection with soil-transmitted helminths including *S. stercoralis* was observed and reported by Rawlins et al. [8, 9] in Jamaica from the hospital-based setting and island-wide surveys.

The prevalence of *S. stercoralis* infection in Jamaica was most recently evaluated in a focused community-based survey in 1995 and reported as 24.2% using an in-house ELISA for detection of parasite-specific IgG antibodies and 3.5% using coprological examination [3]. Prior, Rawlins et al. [9] reported the prevalence of *S. stercoralis* infection as 0.3% based on a country-wide prospective study of intestinal parasites in Jamaica. Therefore, the public health significance of the parasite is currently unknown. This study used an ELISA for the detection of parasite-specific IgG to determine the seroprevalence of *S. stercoralis* in a selected population in Jamaica and set the stage for detailed coprological studies, as necessary.

## 2. Materials and Methods

### 2.1. Study Design and Sample Selection

The study was performed on 311 archived serum samples in the Department of Microbiology, University of the West Indies, Mona, in Kingston, Jamaica. The samples were submitted to the department between 2014 and 2015 for investigation of mosquito-borne viral illnesses from the four Regional Health Authorities of the Ministry of Health and Wellness (MOHW), across the country of Jamaica. The parishes of Jamaica comprising each Regional Health Authority are outlined below:

• The North East Regional Health Authority (NERHA) is comprised of three rural parishes: Saint Ann, Saint Mary, and Portland.
• The South East Regional Health Authority (SERHA) is mixed urban and rural comprised of the parishes: Kingston and Saint Andrew, Saint Catherine, and Saint Thomas.
• The Southern Regional Health Authority (SRHA) is comprised of rural parishes: Saint Elizabeth, Manchester, and Clarendon.
• The Western Regional Health Authority (WRHA) is comprised of the mixed urban and rural parishes: Saint James, Trelawny, Hanover and Westmorland.

The patient data including age, sex, diagnosis, and geographic location of sample submission were retrieved from the laboratory information system or patient records. Serum samples were stored at −20°C and assay for IgG to *S. stercoralis* was performed within 2 hours of thawing using the AccuDiag *Strongyloides* IgG ELISA kit (Diagnostic Automation/Cortez Diagnostics, Inc., California, United States of America) according to the manufacturer's instructions. Positive tests are defined by a value greater than 0.2 optical density (OD) units.

### 2.2. Exclusion Criteria

Serum samples of insufficient volume and those showing signs of hemolysis or cloudiness (indicative of microbial growth or high lipid content) were not included in the study.

### 2.3. Ethical Issues

The study protocol was approved by the University of the West Indies (UWI), Mona Campus Ethics Committee (ECP 260, 16/17). To maintain anonymity, samples were reassigned data entry numbers upon enrollment to the study and de-identified.

### 2.4. Statistical Analysis

Data analysis was performed using Stata version 13.

## 3. Results

There was an almost equal number of males and females (159 versus 152), and the median age of the study group was 10 years with a range from 1 month to 92 years. Most samples originated from the South East Regional Health Authority (*n* = 175; 56.27%), which is the most urbanized Regional Health Authority of the Jamaican MOH. This was followed by the Western Regional Health Authority (*n* = 49; 15.76%), then the Southern Regional Health Authority (*n* = 45; 14.47%) and the North East Regional Health Authority (*n* = 42; 13.50%).

The seroprevalence of *S. stercoralis* in the samples tested based on positive IgG serology was 15.43% (48/311). The seroprevalence among females (16.45%; 25/152) was not statistically significantly different than among males (14.47%; 23/159).

### 3.1. Prevalence of *S. stercoralis* IgG Antibodies According to Age

**Figure 1:**
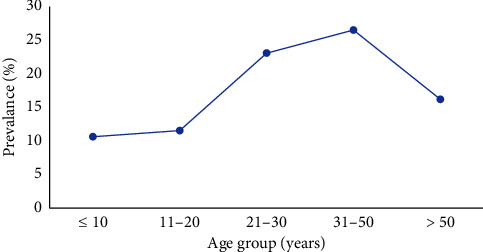
Age-related seroprevalence curve of *S. stercoralis* IgG antibodies.

**Figure 2:**
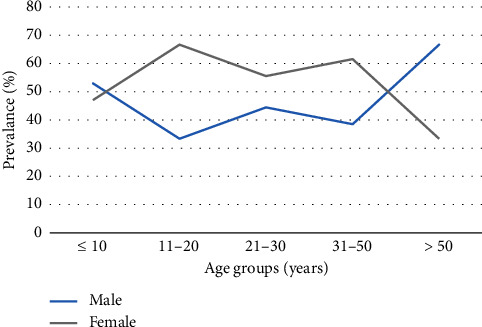
Age-related seroprevalence profile by sex of *S. stercoralis*.

Seropositivity to *S. stercoralis* was detected in patients as young as ≤ 10 years of age (10.63%; 17/160) with a peak in middle adulthood at age 31–50 years (26.53%; 13/49) followed by a decline to 16.22% in those ≥ 50 years of age (Figure 1). Seropositivity to *S. stercoralis* occurred more among males than females at extremes of age (≤ 10 years and > 50 years) (Figure 2).

### 3.2. Seroprevalence of *S. stercoralis* IgG Antibodies in School-Aged Children (5–16 Years)

**Figure 3:**
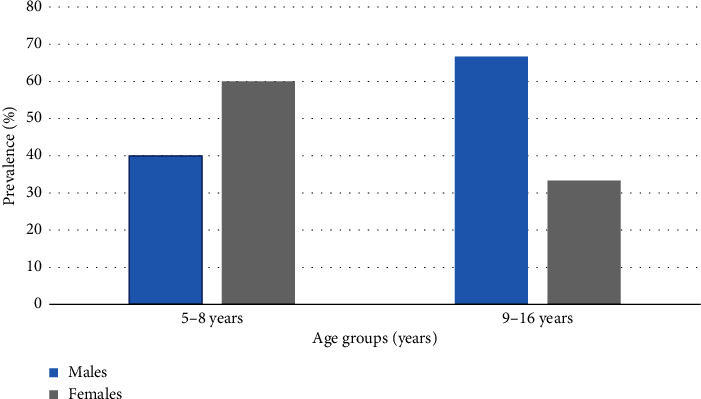
Seroprevalence of *S. stercoralis* IgG antibodies in school age children (5–16 years) according to sex.

The seroprevalence of *S. stercoralis* IgG antibodies in younger school children aged 5–8 years was not significantly different from that of older school children aged 9–16 years (*χ*^2^ = 0.0422; *p*=0.837). However, seroprevalence was higher among females than males in the younger school age group of 5–8 years (Figure 3). Conversely, seroprevalence was higher among males than females in the older school age group of 9–16 years (Figure 3).

Geographically, the seropositivity of *S. stercoralis* IgG antibodies was represented more frequently in the Regional Health Authorities of rural Jamaica, namely, the North East Regional Health Authority (NERHA) and the Southern Regional Health Authority (SRHA) at rates of 33.33% (*n* = 14/42) and 20% (*n* = 9/45), and least within the urban South East Regional Health Authority (9.71%; 17/175).

## 4. Discussion

The seroprevalence of *S. stercoralis* IgG antibodies was determined to be 15.43% in this study. Prior to this, Lindo et al. [3] reported the seroprevalence of *S. stercoralis* as 24.2% from a community-based surveillance study, using an in-house ELISA. The study of Lindo et al. [3] may have been an overestimate, as the sampling was done at endemic foci identified by a known infected index case, and clustering of infections has been previously reported [10]. On the other hand, the true prevalence may have declined due to improvements in the standard of living in Jamaica over the past 30 years. Rawlins et al. [9] reported findings of a decline in soil-transmitted helminths in Jamaica from a country-wide prospective study in which the prevalence of *S. stercoralis* infection was 0.3% [9]. This would mirror the pattern seen among other soil-transmitted helminths within other Caribbean communities as also reflected by hospital records in the region.

The seroprevalence of *S. stercoralis* between males and females was not significantly different in this study which suggests that opportunities for exposure occurred at equal rates in either sex. Although not demonstrated in this study, there have been previous reports of a male predominance in the prevalence of *S. stercoralis* infection [11–14]. Rawlins et al. [12], in a prevalence study for the detection of *S. stercoralis* infection among patients attending UHWI between 1980 and 1981 reported 64% of cases occurring in males. Similarly, a systematic review by Barroso et al. [13] assessed the endemic cases of *S. stercoralis* in Spain and reported a higher infection in men with attribution to dedication in agriculture.

The pattern of exposure to *S. stercoralis* by age is indicative of the acquisition of infection occurring at an early age. This is a typical trend observed from surveys of soil-transmitted helminths [3, 15, 16]. In this study, peak seropositivity occurred middle in adulthood (31–50 years). In Jamaica, young children have more frequent interactions with soil by way of participation in outdoor recreational activities and/or the practice of walking outside in the absence of shoe coverage [3, 15]. Such activities often present ideal opportunities for soil-based transmission by the infective filariform (L3) larvae of *S. stercoralis*. However, in this study, seropositivity to *S. stercoralis* among school-aged children 5–16 years old was not found to be significantly different between younger children of age 5–8 years and older children of age 9–16 years. In middle adulthood, a peak in the seropositivity of *S. stercoralis* IgG antibodies was more indicative of autoinfection, and less likely due to newly acquired parasitic disease [5].

The age-prevalence profile of *S. stercoralis* by sex in this study revealed a male predominance of seropositivity occurring at extremes of age (Figure 3). Conversely, a female predominance in the seropositivity of *S. stercoralis* IgG antibodies was apparent in grade-schoolers (5–8 years) (Figure 3). This observed trend in the age-related seropositivity of *S. stercoralis* IgG antibodies by sex is likely due to the previously mentioned participation in recreational, domestic, and/or occupational activities (such as lack of shoes outdoors and improper toilet facilities), which promote opportunities for soil-based transmission from soil-bearing infective larvae [17, 18].

Serology has been previously employed in settings highly endemic for *S. stercoralis* such as countries of Latin America, to elucidate the seroepidemiology of *S. stercoralis* infections and highlight underestimation of disease burden [7, 19, 20]. In rural Peru, as an example, seropositivity to *S. stercoralis* was estimated at 72% using serology; however, an infection rate of 8.7% was determined using stool-based laboratory methods [7]. A similar disparity between seropositivity and a conventional coprological microscopic technique in the detection of *S. stercoralis* was also reported by Lindo et al. [3] from a community-based survey in Jamaica. These findings are also consistent with a prospective study examining the diagnostic accuracy of urine-based assay for *S. stercoralis* with coprological and serological methods in Thailand, where the prevalence rates for urine-based assay (68.5%) and serum ELISA (65.8%) were comparable, yet prevalence rate for the coprological method was 27.5%, revealing less sensitivity results to *S. stercoralis* when compared to the other methods [21]. In this study, the prevalence of infection based on stool assessment was found to be 3.5%, whilst by ELISA seropositivity was 24.2%. This further emphasizes the importance of using *S. stercoralis* specific diagnostic methods to reliably assess the prevalence of *S. stercoralis* infection in endemic regions. The sensitivities of ELISA in the detection of IgG antibodies to *S. stercoralis* antigens have been reported as high as 90%–92% [5, 22, 23]. IgG antibodies are the most abundant antibody type produced against the *S. stercoralis* larval antigen [5]; hence, its use as a serological marker may be indicative of current or previous exposure. The rapid AccuDiag *Strongyloides* IgG ELISA kit by Diagnostic Automation/Cortez Diagnostics Inc., lists specificity and sensitivity as 100%, albeit based on comparison to another commercially available ELISA.

The seropositivity of *S. stercoralis* IgG antibodies was higher in the Regional Health Authorities of rural Jamaica, the parishes comprising the Regional Health Authorities of NERHA and SRHA are of rural, agriculturally based communities and wetland areas of Jamaica. These parishes represent prime environmental conditions toward the maintenance of the free-living (heterogenic) phase of the *S. stercoralis* life cycle, which facilitates the development of the relevant larval forms crucial to the parasitic (homogenic) phase of the life cycle. The latter stages of larval development is a key to establish infection inside the human host [23, 24]. Occupational, domestic, and recreational activities within these parishes are primarily agriculturally focused, which thus enhances and sustains the soil-based transmission of infective filariform larvae of *S. stercoralis* [4–6, 25, 26].

The parasite-specific IgG ELISA used in the study does not discern between acute or current infections; hence no comments could have been made regarding disease activity or severity. The results may be interpreted as an indication of recent or past infection with *S. stercoralis* which is useful for epidemiological studies but not clinical diagnosis. Thirdly, there is the potential for false positives results based on of cross-reactivity with other helminths such *as Trichuris trichiura*, *Ascaris lumbricoides*, and hookworms. The findings cannot be extrapolated to the general population of Jamaica because of the biased nature of sampling (persons with fever).

## 5. Conclusion

The seroprevalence of *S. stercoralis* IgG antibodies in this collection of serum samples from Jamaica was 15.43% using a rapid ELISA assay for the detection of IgG antibodies. There appeared to be higher seropositivity rates from rural areas of the country. These results illustrate the importance of *S. stercoralis* as a parasite of humans in Jamaica and suggest the need for additional cross-sectional studies using a combination of stool microscopy and serology to elucidate the epidemiology of this soil-transmitted helminth and establish its public health significance. The findings of this study highlight the usefulness and convenience of a rapid serological technique in detecting IgG antibodies to *S. stercoralis*, as an adjunct to existing laboratory methods for screening and investigation.

## Data Availability

The data that support the findings of this study are available on request from the corresponding author. The data are not publicly available due to privacy or ethical restrictions.
